# Patient-Derived Avatar Mouse Model to Predict the Liver Immune Homeostasis of Long-Term Stable Liver Transplant Patients

**DOI:** 10.3389/fimmu.2022.817006

**Published:** 2022-03-28

**Authors:** Soon Kyu Lee, Min-Jung Park, Jeong Won Choi, Jin-Ah Baek, Se-Young Kim, Ho Joong Choi, Young Kyoung You, Jeong Won Jang, Pil Soo Sung, Si Hyun Bae, Seung Kew Yoon, Jong Young Choi, Mi-La Cho

**Affiliations:** ^1^ Division of Hepatology, Department of Internal Medicine, Incheon St. Mary's Hospital, College of Medicine, The Catholic University of Korea, Seoul, South Korea; ^2^ The Rheumatism Research Center, Catholic Research Institute of Medical Science, College of Medicine, The Catholic University of Korea, Seoul, South Korea; ^3^ Department of Surgery, Seoul St. Mary’s Hospital, College of Medicine, The Catholic University of Korea, Seoul, South Korea; ^4^ Division of Hepatology, Department of Internal Medicine, Seoul St. Mary's Hospital, College of Medicine, The Catholic University of Korea, Seoul, South Korea; ^5^ Division of Hepatology, Department of Internal Medicine, Eunpyeong Se. Mary’s Hospital, College of Medicine, The Catholic University of Korea, Seoul, South Korea

**Keywords:** liver transplantation, avatar model, inflammation, fibrosis, immune homeostasis

## Abstract

Although rejection or tolerance can occur in liver transplantation (LT) patients, there are no reliable non-invasive methods for predicting immune homeostasis. In this study, we developed a humanized mouse model to predict liver immune homeostasis in patients who underwent LT. The patient-derived avatar model was developed by injecting peripheral blood mononuclear cells from healthy controls (HCs) or LT patients with stable, rejection, or tolerance into NOD.Cg-*Prkdc* ^scid^ *IL2rg* ^tm1Wjl^/SzJ (NSG) mice, followed by injection of human hepatic stellate cells and Carbone tetrachloride (CCl_4_). After 7 weeks, the patient’s T-cell engraftment and liver inflammation in the avatar model were evaluated and compared with the liver histology of LT patients. Changes in liver inflammation following treatment with tacrolimus and/or biguanide derivatives were also examined. The C-X-C Motif Chemokine Receptor 3 (CXCR3)-dependently engrafted patient T cells led to differences in liver inflammation in our model according to the status of LT patients. The livers of avatar models from rejection patients had severe inflammation with more T helper 17 cells and fewer regulatory T cells compared to those of models from tolerance and HCs showing only mild inflammation. Moreover, our model classified stable post-LT patients into severe and mild inflammation groups, which correlated well with liver immunity in these patients. Our models revealed alleviation of inflammation after combination treatment with tacrolimus and biguanide derivatives or monotherapy. Consequently, using our new patient-derived avatar model, we predicted liver immune homeostasis in patients with stable LT without biopsy. Moreover, our avatar model may be useful for preclinical analysis to evaluate treatment responses while reducing risks to patients.

## Introduction

Liver transplantation (LT) is an eventual treatment for patients with end-stage liver disease and hepatocellular carcinoma. However, the use of long-term immunosuppressants (ISs) is associated with risks of cancer, cardiovascular events, and renal complications (1, 2). Even after long-term maintenance, the reduction or withdrawal of IS carries a risk of rejection in some patients, whereas others may show graft tolerance.

In the long term, patients with normal liver function tests after LT exhibit a risk of rejection, particularly in cases when ISs are tapered, and not all patients can achieve minimization of IS or tolerance. Liver function tests alone cannot definitively represent liver immunity in LT patients. To overcome this limitation, researchers have attempted to document the immune status of patients with LT by analyzing peripheral blood mononuclear cells (PBMCs). Recent studies revealed the importance of regulatory T (Treg) and T helper 17 (Th 17) cells in both rejection and tolerance in LT patients (3, 4). An early reduction in the number of Treg cells and an increase in the number of Th17 cells after LT are associated with acute rejection; reciprocally, an increase in the number of Treg cells and a decrease in the number of Th17 cells during tapering of ISs are related to successful tolerance in patients with LT (5–7). However, as shown in our previous study, the possibility of minimization or tolerance represented as an increase in the Treg/Th17 cell ratio could be differentiated only after and not before tapering of ISs (6).

Whether liver immunity is balanced, particularly in long-term stable patients, and whether a patient is likely to minimize ISs successfully or experience rejection before adjusting the IS dosage are unclear. Analysis of blood tests and PBMCs may not be sufficient for predicting liver immune microenvironments under the effects of ISs. Liver biopsy remains the gold standard for assessing liver immunity and guiding IS management (3). Recently, *in situ* multiplex immunofluorescence analysis of renal allograft histology was introduced to quantify the inflammatory burden and characterize the alloimmune response in patients with kidney allograft rejection (8). Nevertheless, there are limitations to performing biopsy, specifically in stable patients, because of its invasiveness and potential risk of complications (9). Therefore, non-invasive biomarkers or models for predicting liver immunity in LT patients, particularly in stable patients, must be developed.

Recent advances in the development of mice with human immune systems (humanized mice), which are immunodeficient mice engrafted with human cells or tissues, have led to various preclinical studies of the molecular pathways and response to chemotherapy in cancer human immune system models (10, 11). In transplant immunology, humanized mouse models with allografts of skin, islets, cardiac tissues, and pluripotent stem cells have been developed and used to study human immune responses (12). However, there are no reliable models that can reflect the real liver immunity of LT patients and give clinicians the opportunity to assess liver immunity and guide future treatment plans. Furthermore, preclinical methods are needed to examine and predict treatment responses to various drugs in LT patients without the risk of complications in these patients.

Herein, we developed an avatar mouse model for predicting the status of liver inflammation in LT patients by using PBMCs from these patients. This avatar mouse model enables the classification of LT patients reflecting real liver inflammation without liver biopsy. Moreover, our avatar model shows potential as a preclinical model for evaluating treatment responses in various drugs without putting patients at risk.

## Materials and Methods

### Patients

A total of 41 patients were prospectively enrolled from a single LT clinic at Seoul St. Mary Hospital between January 2018 and October 2020. PBMCs and, if possible, the liver histopathological status of each patient were collected, analyzed, and used to generate a patient-derived avatar model. Among them, 12 patients were in the rejection group, three were in the tolerance group, and the remainder (n = 26) were in the stable group. Rejection was defined according to the Banff criteria (13, 14), and tolerance was defined as patients who withdrew ISs safely and were stable for more than 1 year (3). The stable group included long-term post-LT patients (>5 years from LT) administered ISs with normal liver function. Healthy controls (HCs; n = 7) without any medical diseases were also recruited. Written informed consent was obtained from all included patients. This study was approved by the institutional review board of Seoul St. Mary’s Hospital (KC19TESI0612) and performed in accordance with the Declaration of Helsinki.

### Mice

Female NOD/scid/IL-2Rγ^−/−^ mice (NOD.Cg-*Prkdc* ^scid^ *IL2rg* ^tm1Wjl^/SzJ; abbreviated as NSG) at 6–8 weeks of age were obtained from The Jackson Laboratory (Bar Harbor, ME, USA). The mice were maintained under specific pathogen-free conditions in an animal facility and given autoclaved food and water. A high efficiency particulate air (HEPA) filter system was used to exclude bacteria and viruses from the air in the facility. The protocols used in this study were approved by the Animal Care and Use Committee of the Catholic University of Korea (CUMC- 2020-0355-02).

### Animal Experimental Design

**Figure 1:**
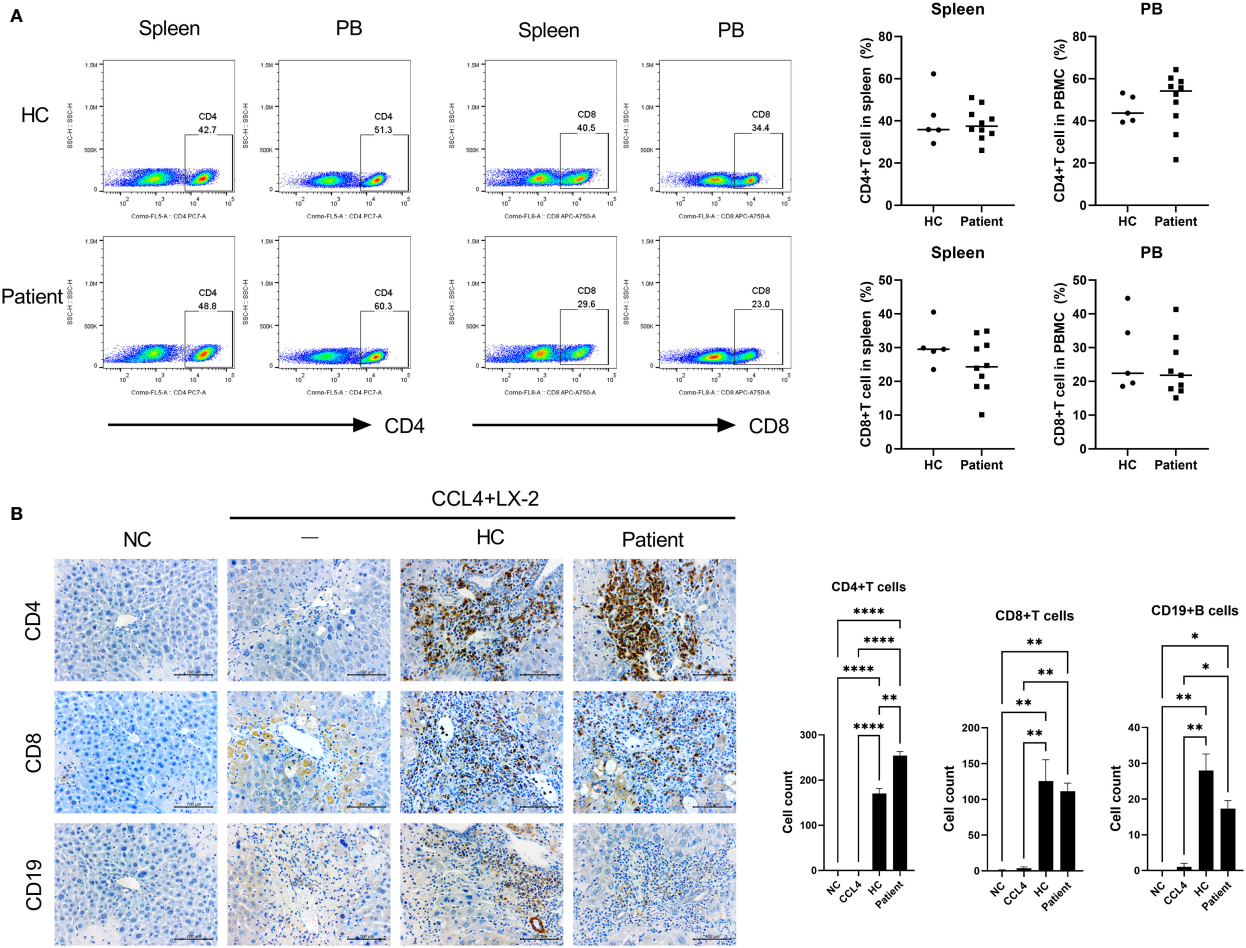
Generation of patient-derived avatar models in LT patients. **(A)** To develop the humanized mouse model from patient-derived PBMC, PBMCs were isolated from whole-blood samples obtained from LT patients or HCs. Freshly isolated PBMCs (5 × 10^6^) were injected intravenously into NSG mice. At 1 day after intravenously transplanting hHSCs to engraft, mice were injected with CCl_4_ twice weekly at various time points as indicated. At 49 days after induction of humanized mice, recipient spleens and peripheral blood were collected to analyze the engraftment levels of human CD4^+^ and CD8^+^ T-cell populations in the spleen and blood by using flow cytometry. **(B)** Histologic analysis of infiltration of human CD4^+^ and CD8^+^ T cells and CD19^+^ B cells in mouse liver tissue by immunohistochemistry staining. Data were obtained from three independent experiments, and values are represented as the mean ± SEM. LT, liver transplantation; PBMC, peripheral blood mononuclear cell; NC, negative control; HC, healthy control; hHSC, human hepatic stellate cell. *P < 0.05; **P < 0.01; ****P < 0.0001.

To develop the patient-derived model, freshly isolated human PBMCs (5 × 10^6^/mouse) from HCs or LT patients were intravenously injected into NSG mice. To emphasize the immune response of T-cells that can lead to enhanced liver inflammation and fibrosis, infusion of human hepatic stellate cells (hHSCs) and CCl_4_ was performed. Specifically, NSG mice were intravenously injected with hHSCs [LX-2 cell line from Merck Millipore (Darmstadt, Germany)] on day 1 followed by intraperitoneal injections with a low dose of CCl_4_ [6 μl in corn oil 100 μl/mice (0.3 ml/kg)] twice per week for 7 weeks (**Figure 1A**). This avatar model was compared with other experimental designs as follows: type 1, only injected with PBMCs; type 2, injected with PBMCs followed by injections with CCl4; and type 3, injected with PBMCs followed by injections with hHSCs (**Supplementary Figure S1**).

In the drug treatment groups, at 4 weeks after induction of humanized mice, recipient mice were administered SD282 (N-ethyl-N-[4-fluorophenyl] biguanide derivatives, 50 mg/kg), biguanide derivatives, and/or tacrolimus (0.5 mg/kg) every 3 weeks. Control mice were administered the vehicle control (dimethyl sulfoxide diluted in saline) in the same manner as the treatment group.

### Measurement of Serum AST and ALT Levels in Avatar Models

Serum samples were prepared by centrifugation (8,000 rpm for 5 min) and analyzed for biochemical parameters. The serum aspartate aminotransferase (AST) and alanine aminotransferase (ALT) levels were determined using commercial kits (Asan Pharmaceutical Co., Hwangseong-gi, Gyeonggi-do, Republic of Korea).

### Flow Cytometry and Enzyme-Linked Immunosorbent Assay

For flow cytometric analysis, whole spleens were macerated into single-cell suspensions of splenocytes by using the frosted ends of two microscope slides. Cell suspensions were passed through a cell strainer (40 µm) (Corning, Inc., Corning, NY, USA) to eliminate clumps and debris, followed by lysis of red blood cells with Ammonium–Chloride–Potassium (ACK) lysis buffer. Mononuclear cells from the peripheral blood and spleen of the avatar models and PBMCs from patients were stained with various combinations of fluorescent antibodies against human CD4, CD8, CD19, CD25, CD45RA, chemokine receptor 7 (CCR7), Interleukin 17 (IL-17), Interferon (IFN)-γ, and FoxP3 (**Supplementary Table S1**). Prior to intracellular staining, the cells were restimulated for 4 h with phorbol myristate acetate (25 ng/ml) and ionomycin (250 ng/ml) in the presence of GolgiSTOP (BD Biosciences, San Diego, CA, USA). Intracellular staining was performed using a kit (eBioscience, San Diego, CA, USA) according to the manufacturer’s protocol. Flow cytometry was performed on a FACSCalibur instrument (BD Biosciences). We defined Treg cells as CD25^+^FoxP3^+^CD4^+^T cells, Th1 cells as IFN-γ^+^CD4^+^T cells, Th17 cells as IL-17^+^CD4^+^T cells including IL-17^+^pSTAT3^+^CD4^+^T cells, central memory (CM)-Th17 cells as IL-17^+^ CD4^+^CD45RO^+^CD45RA^-^CCR7^+^ cells, and effector memory (EM)-Th17 cells as IL-17^+^ CD4^+^CD45RO^+^CD45RA^-^CCR7^-^ cells (15–18).

### Histological Analysis and Chemotaxis Experiments

At 7 weeks after cell transfer into NSG mice, the liver tissues were harvested, cryoembedded, and sectioned. The tissue specimens were fixed in 10% formalin buffer and embedded in paraffin. At least three slices (6-μm-thick) were prepared from each sample and stained with hematoxylin and eosin (H&E), Masson’s trichrome (MT), and Sirius red. The histological scores were blindly examined using an established scoring system including the Histology Activity Index, Rejection Activity Index, and fibrosis, without knowing the clinical status of each model, and the results were compared between the rejection and tolerance groups (13, 19). The liver histology of patients (n = 25) was also evaluated and compared to that of their own avatar mice to verify this avatar model.

For immunohistochemistry (IHC) staining, the sections were stained with antibodies against human CD4, CD8, CD19, CK-19, IL-17, and FoxP3 (**Supplementary Table S2**). For chemotaxis experiments, the sections were stained with several chemokine ligands (CXCL9, CXCL10, CXCL11, CCL2, and CCL3) and chemokine receptors (CXCR3 and CCR2) overnight at 4°C, followed by addition of a biotinylated secondary antibody with a streptavidin peroxidase mixture for 1 h (Thermo Fisher Scientific, Waltham, MA, USA).

Migration assays were performed in a 24-well Transwell unit with 3-μm pores (Corning Costar, Cambridge, MA, USA). Th17 cells at a density of 2 × 10^5^ cells in 100 μl of medium (1% fetal bovine serum) were seeded into the upper chamber of the Transwell assembly. LX-2 cells (1 × 10^5^) in the lower chamber containing 600 μl of medium were used to attract human Th17 cells in a classical chemotaxis assay. After incubation at 37°C and 5% CO_2_ for 24 h, the upper surface of the membrane was scraped gently to remove non-migrating cells and washed with phosphate-buffered saline. Migrated cells were fixed in 4% paraformaldehyde for 15 min and stained with 0.5% crystal violet for 10 min.

### Confocal Microscopy and Immunostaining

Mouse and human liver tissue cryosections (7-μm-thick) were fixed in 4% (v/v) paraformaldehyde and stained using fluorescein isothiocyanate-, phycoerythrin-, PerCP-Cy5.5-, or allophycocyanin-conjugated monoclonal antibodies against human CD4, CD25, Foxp3, IL-17, CXCR3, phophoSTAT3 (Tyr 705), and 4',6-diamidino-2-phenylindole (DAPI) (Invitrogen, Carlsbad, CA, USA) (**Supplementary Table S3**). After overnight incubation at -4°C, the stained sections were visualized by confocal microscopy (LSM 510 Meta; Zeiss, Göttingen, Germany).

### Statistical Analysis

The patients’ baseline characteristics are presented as the mean ± standard deviation or counts (percentage), as appropriate. Experimental data are expressed as the mean ± standard error of the mean (SEM). Differences between groups were analyzed using Student’s t-test or Mann–Whitney U test for categorical variables and chi-square test or Fisher’s exact test for continuous variables, as appropriate. One-way analysis of variance followed by Bonferroni’s *post-hoc* test or Kruskal–Wallis H test was used to compare differences between three or more groups where appropriate. Statistical significance was set at *P* < 0.05. All statistical analyses were performed using Prism (standard version 5.01; GraphPad, Inc., San Diego, CA, USA) and R version 4.0.4 (http://cran.r-project.org; The R Project for Statistical Computing, Vienna, Austria).

## Results

### Baseline Characteristics of the Study Population

The mean age of patients was 58.7 years, and 32 patients (78.0%) were men. Among the 41 patients, 38 (78.0%) underwent living donor LT, with liver cirrhosis (n = 24, 58.5%) and hepatocellular carcinoma (n = 9, 22.0%) as the major reasons for LT. Most patients (n = 29, 70.7%) were treated with tacrolimus at a mean dose of 2.1 mg/day. The mean time from LT in all groups was more than 10 years (10.9 ± 4.6 years). The rejection group had significantly higher levels of AST, ALT, and alkaline phosphatase than those of the other groups. The tolerance group had normal liver functions, including the levels of AST, ALT, and alkaline phosphatase, and marginally older age (63.7 years) with a longer time from LT (13.6 years) compared to the other groups (**Table 1**).

**Table 1:** Baseline characteristics of the entire population.

Variables	Total (N = 41)	Rejection group (n = 12)	Stable group (n = 26)		Tolerance group (n = 3)	*P*-value
			MIS group (n = 15)	SIS group (n = 11)		
Age, years	58.7 ± 8.0	57.3 ± 9.0	57.1 ± 7.9	60.9 ± 6.7	63.7 ± 9.2	0.539
Male sex (n, %)	32 (78.0%)	10 (83.3%)	11 (73.3%)	8 (72.7%)	3 (100%)	0.859
LDLT	38 (92.7%)	12 (100%)	13 (86.7%)	10 (90.9%)	3 (100%)	0.565
Cause of LT						
- LC/HCC/ALF	24 (58.5%)/9 (22.0%)/8 (19.5%)	5 (41.7%)/1 (8.3%)/6 (50.0%)	9 (60.0%)/4 (26.7%)/2 (13.3%)	8 (72.7%)/3 (27.3%)/0 (0.0%)	2 (66.7%)/1 (33.3%)/0 (0.0%)	0.1
- HBV/others	25 (61.0%)/16 (39.0%)	1 (8.3%)/11 (91.7%)	11 (73.3%)/4 (26.7%)	10 (90.9%)/1 (9.1%)	3 (100.0%)/0 (0.0%)	<0.001
Type of IS						0.049
- Tacrolimus	29 (70.7%)	12 (100.0%)	9 (60.0%)	8 (72.7%)	–	
- Cyclosporine	9 (22.0%)	0 (0.0%)	6 (40.0%)	3 (27.3%)	–	
Dose of IS (mg)						
- Tacrolimus	2.1 ± 1.0	2.3 ± 1.2	1.9 ± 1.2	2.1 ± 0.6	–	0.671
- Cyclosporine	61.1 ± 25.3	–	66.7 ± 30.3	50.0 ± 0.0	–	0.762
Level of IS (ng/ml)						
- Tacrolimus	4.5 ± 2.5	4.8 ± 2.8	3.2 ± 2.4	5.4 ± 1.9	–	0.744
- Cyclosporine	54.0 ± 33.3	–	64.0 ± 36.6	34.1 ± 13.4	–	0.225
AST (U/L)	70.4 ± 186.9	185.1 ± 326.7	24.1 ± 6.8	22.0 ± 5.6	20.3 ± 1.5	0.003
ALT (U/L)	94.9 ± 273.6	268.8 ± 474.3	26.7 ± 17.1	20.0 ± 13.9	14.0 ± 3.0	0.002
ALP (mg/dl)	82.9 ± 35.8	101.0 ± 41.7	80.2 ± 23.7	63.2 ± 14.0	96.7 ± 82.0	0.048
r-GTP (mg/dl)	117.5 ± 125.9	231.6 ± 145.7	92.9 ± 96.5	47.5 ± 35.1	28.0 ± 9.5	<0.001
Total bilirubin (mg/dl)	2.7 ± 7.9	6.7 ± 14.2	1.0 ± 0.4	1.2 ± 0.7	0.9 ± 0.8	0.588
Albumin (g/dl)	4.2 ± 0.5	3.9 ± 0.6	4.2 ± 0.3	4.4 ± 0.3	4.2 ± 0.5	0.087
PT INR	1.0 ± 0.1	1.1 ± 0.2	1.0 ± 0.0	1.0 ± 0.1	1.1 ± 0.1	0.253
Platelet (×10^9^/L)	180.7 ± 69.2	151.7 ± 47.9	181.6 ± 60.0	202.5 ± 64.1	193.7 ± 159.8	0.091
Post-LT, years	10.9 ± 4.6	9.1 ± 3.8	11.2 ± 4.9	11.6 ± 5.0	13.6 ± 4.6	0.096

MIS, mild inflammation in stable; SIS, severe inflammation in stable; LDLT, living donor liver transplantation; LT, liver transplantation; LC, liver cirrhosis; HCC, hepatocellular carcinoma; ALF, acute liver failure; IS, immunosuppressant; AST, aspartate aminotransferase; ALT, alanine aminotransferase; ALP, alkaline phosphatase; r-GTP, gamma glutamyl transferase; INR, international normalized ratio; PT INR, Prothrombin time international normalized ratio.

### Generation of a Patient-Derived Avatar Model

We generated a patient-derived avatar model as described in the *Materials and Methods* section. At 7 weeks after injection with human PBMCs, we evaluated the engraftment levels of human CD4^+^ and CD8^+^ T cells in the spleen and PBMCs of the avatar models. In both avatar models injected with PBMCs from HC and LT patients, CD4^+^ (40%–60%) and CD8^+^ (10%–20%) T cells were engrafted well without significant differences (**Figure 1A**). In histological evaluation of avatar model livers on day 49 after human PBMC injection, CD4^+^, CD8^+^ T, and CD19^+^ B cells were engrafted well in both avatar models injected with PBMCs from HC and LT patients and significantly differed from the negative controls (**Figure 1B**).

After engraftment of human CD4^+^ cells, CD8^+^ T cells, and CD19^+^ B cells in our avatar model, inflammation and fibrosis were induced in the liver. However, in the other type of experimental design injected with PBMCs, minimal inflammation was observed. The other types injected with PBMCs followed by CCl_4_ or hHSCs also showed less inflammation compared to our avatar models, although these types showed greater inflammation than the model only injected with PBMCs (**Supplementary Figure S1**). None of the experimental designs, including our avatar model, showed significant inflammation in the spleen or gut (data not shown).

### Verification of Patient-Derived Avatar Model by Comparing Liver Transplantation Patients With Their Avatar Models: Immunological Classification of Stable Liver Transplantation Patients

**Figure 2:**
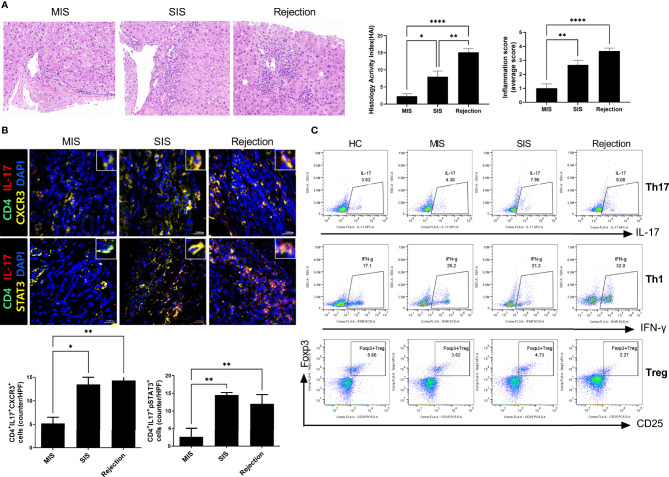
Liver histologic and T-cell subset analysis of LT patients. **(A)** Representative liver histology of LT patients stained with H&E. **(B)** Representative confocal images of CD4-IL-17, CXCR3, and STAT3 in liver histology of LT patients. The number of positive cells was counted in five independent liver tissues. **(C)** Plots from representative experiment show the frequencies of IL-17^+^ (Th17), IFN-γ^+^ (Th1), and CD25^+^FoxP3^+^ (Treg) cells among CD4^+^ T cells in PBMCs of LT patients. Values represent the mean ± SD for six mice per group from at least three independent experiments. ^*^ *P* < 0.05, ^**^ *P* < 0.01, ^****^ *P* < 0.0001. H&E, hematoxylin and eosin; LT, liver transplantation; MIS, mild inflammation in stable; SIS, severe inflammation in stable; HC, healthy control; PBMC, peripheral blood mononuclear cell.

We used our generated patient-derived avatar models to evaluate and compare inflammation in LT patients and their avatar models by analyzing immune markers and histology. First, we performed histology analysis of the patients to determine their long-term stability and rejection after staining with H&E (**Figure 2A**). Interestingly, although all long-term stable patients had normal liver function, some patients (n = 11) showed high levels of inflammation (SIS group, severe inflammation in the stable group) comparable to those in the rejection group; however, other patients (n = 15) showed only mild inflammation (MIS group, mild inflammation in the stable group). We further evaluated the phenotypes of the inflammatory cells infiltrating the liver in each group. As shown in **Figure 2B**, infiltration of CD4^+^IL-17^+^CXCR3^+^ T cells and CD4^+^IL-17^+^pSTAT3^+^ T cells was significantly higher in the rejection and SIS groups than that in the MIS group. Similarly, analysis of T-cell subsets in PBMCs of patients from the three groups showed that Th17 cells were significantly increased, whereas Treg cells were decreased in the rejection and SIS groups compared to those in the MIS group (**Figure 2C** and **Supplementary Figure S2**).

**Figure 3:**
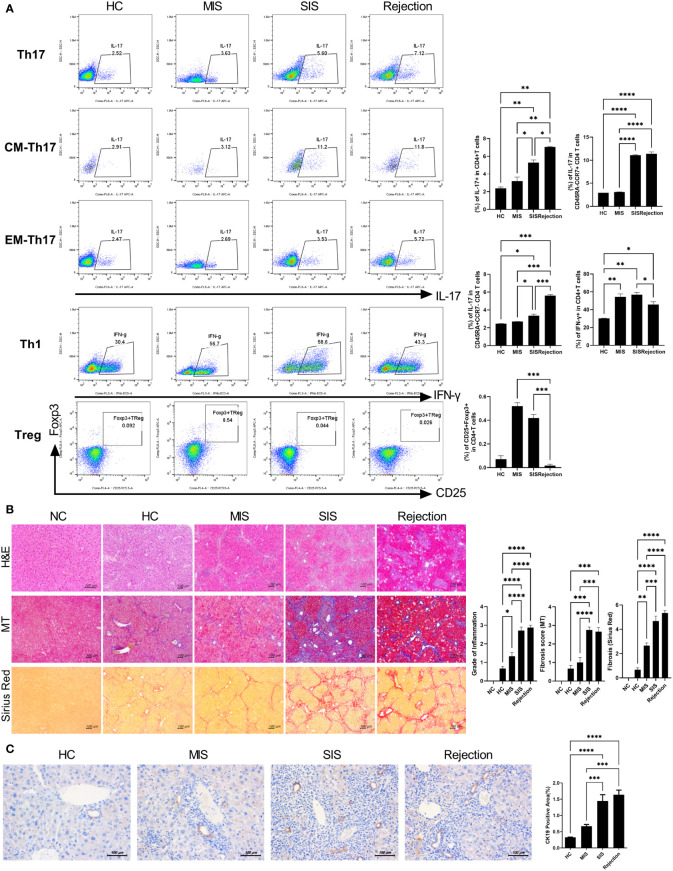
Characterization of patient-derived avatar models by immune profiling of human lymphocytes and analysis of liver histology in the model. **(A)** Representative flow cytometric plot expressing the proportion of CD4^+^ T-cell subtypes in the spleen of each humanized mouse group. At 7 weeks after induction of humanized mice, the engraftment levels of human T-cell subset in the spleen of each patient-derived avatar group were analyzed using flow cytometry. The percentage of each cell population among CD4^+^ T cells is shown in the right upper panel. **(B)** Representative liver histology stained with H&E, MT, and Sirius red in paraffin-embedded avatar models. Liver tissue sections were harvested from humanized mice at 7 weeks after adoptive transfer with PBMC preparations from HC or LT patients. Inflammation and fibrosis scores are shown in the right panel. **(C)** Representative images of IHC with CK19 antibodies. Bars indicate the percentages of positive areas per field. Data are the means ± SEM of two replicates. ^*^ *P* < 0.05, ^**^ *P* < 0.01, ^***^ *P* < 0.001, ^****^ *P* < 0.0001. H&E, hematoxylin and eosin; MT, Masson’s trichrome; NC, negative control; HC, healthy control; LT, liver transplantation; IHC, immunohistochemistry; MIS, mild inflammation in stable; SIS, severe inflammation in stable; CM, central memory; EM, effector memory.

To characterize and compare inflammatory cells in the patient-derived avatar models, we first analyzed the engraftment level of human T-cell subsets in the blood and spleen of each patient-derived mouse group at 49 days after injection with human PBMCs (**Figure 3A** and **Supplementary Figure S3**). Similar to the results observed in LT patients, avatar models from stable LT patients (n = 26) demonstrated different severities of inflammation and could be classified into SIS (n = 11) and MIS (n = 15) groups. Th17, central memory Th17, and effector memory cells were significantly increased in the SIS and rejection groups compared to those in the MIS and HC groups. However, Treg cells were decreased in the SIS and rejection groups, and Th1 cells decreased in the rejection group at a similar level in both the MIS and SIS groups. The CD8^+^ T-cell populations did not significantly differ between the MIS and SIS groups, with a mild increase in the rejection group (**Supplementary Figure S4**). Next, we analyzed the histopathology of the liver in each patient-derived avatar mouse. Similar to the results for the blood and spleen of the patient-derived avatar model, histological analysis of each patient-derived avatar group demonstrated severe inflammation and fibrosis in the SIS and rejection groups compared to those in the MIS and HC groups, which correlated well with the liver of their LT patients (**Figure 3B**). The levels of Cytokeratin-19 (CK-19), a marker for liver progenitor cells, hepatocyte differentiation, and bile ductular reaction (20–22), were also significantly increased in the SIS and rejection groups (**Figure 3C**). Based on these results, our model could reflect and predict liver immunity and immune homeostasis in patients and differentiate clinically stable LT patients.

### Subtypes of Infiltrated Inflammatory Cells in the Liver of Avatar Model and Their CXCR3-Dependent Migration Into the Liver of Avatar Model

**Figure 4:**
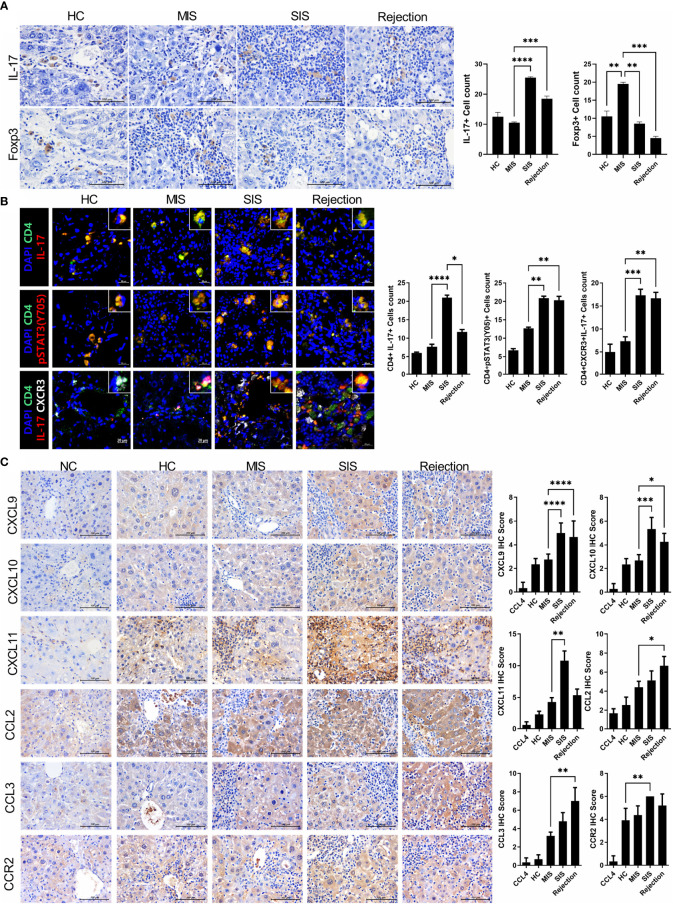
Comparative analysis of infiltrated T-cell subtypes and chemotaxis in the liver of each avatar model group. **(A)** Representative images of IHC with anti-IL-17, FoxP3 antibodies. Bars are percentages of positive areas per field. **(B)** Representative confocal microscopy analysis of Th17 cell localization in liver tissues. Cells were stained with fluorescence-tagged antibodies to determine the counts of CD4^+^IL-17^+^, CD4^+^pSTAT3(tyr 705)^+^, and CD4^+^IL-17^+^CXCR3^+^ cells in liver tissue sections (×100, original magnification). **(C)** Representative images of IHC with anti-CXCL9, CXCL10, CXCL11, CCL2, CCL3, and CCR2 antibodies. Bars indicate the percentages of positive areas per field. Data are the means ± SEM of two replicates. ^*^ *P* < 0.05, ^**^ *P* < 0.01, ^***^ *P* < 0.001, ^****^ *P* < 0.0001. IHC, immunohistochemistry; MIS, mild inflammation in stable; SIS, severe inflammation in stable; NC, negative control; HC, healthy control.

To further verify our model, we identified the type and amount of infiltrated inflammatory cells in the liver of the avatar model. IHC staining with anti-IL-17 and FoxP3 antibodies showed that the rejection group had more IL-17^+^ cells and fewer FoxP3^+^ cells than those in the HC group. Moreover, even in stable LT patients, the SIS group showed many more IL-17^+^ cells and fewer FoxP3^+^ cells than those in the MIS group and comparable to those in the rejection group, whereas the MIS group was similar to the HC group (**Figure 4A**). Similar trends were observed in confocal microscopic analysis of Th17 cells in the liver of each avatar model group. Infiltration of CD4^+^IL-17^+^T cells, CD4^+^IL-17^+^pSTAT3^+^ T cells, and CD4^+^IL-17^+^CXCR3^+^ T cells was significantly higher in the rejection and SIS groups than those in the MIS and HC groups (**Figure 4B**). These findings support that our model can classify patients with stable LT.

Interestingly, the expression of CXCR3, a crucial chemokine for recruiting Th17 cells, was significantly increased in both the SIS and rejection groups, indicating severe inflammation in our models. To recheck the CXCR3-dependent migration of infiltrated inflammatory cells in our model, we investigated the expression of CXCR3 ligands such as CXCL9-11 (**Figure 4C**). The expression of CXCL9-11 was significantly increased in the SIS and rejection groups compared to those in the HC and MIS groups. CCL2-3 and CCR2 expression was also higher in the SIS and rejection groups. Furthermore, in *in vitro* migration analysis, the migration of Th17 cells toward activated LX-2 cells was significantly decreased in anti-CXCR3-treated Th17 cells compared to Th17 cells without anti-CXCR3 antibody (**Supplementary Figure S5**). These results suggest that CXCR3 and its ligands contribute to infiltration of engrafted inflammatory cells into the liver in our patient-derived mouse model.

### Patient-Derived Avatar Model in Tolerant Patients: A Well-Designed Humanized Mouse Model

**Figure 5:**
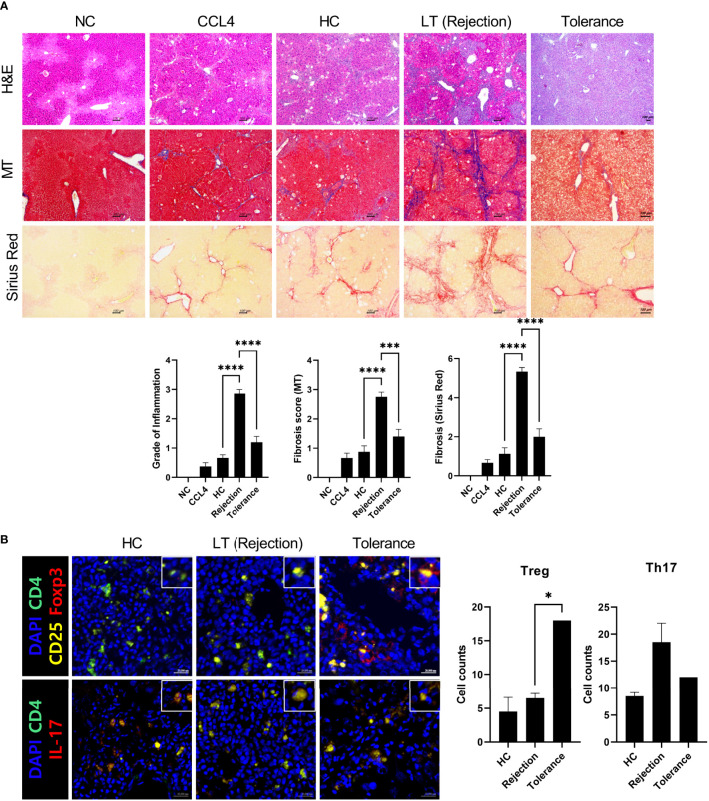
Histologic evaluation of patient-derived avatar models generated from tolerant patients. **(A)** Representative liver histology of avatar models from HC, rejection, and tolerant patients. Liver tissue sections were harvested from humanized mice at 7 weeks after adoptive transfer with PBMC preparations from HC or LT patients (rejection and tolerance) and stained with H&E, MT, and Sirius red in paraffin-embedded avatar model (upper panel). Inflammation and fibrosis scores are shown in the lower panel. **(B)** Representative confocal microscopic analysis for Treg and Th17 cell localization in liver tissues. Cells were stained with fluorescence-tagged antibodies to determine the counts of CD4^+^CD25^+^Fox3^+^ (Treg) and CD4^+^IL-17^+^ (Th17) cells in liver tissue sections (×100, original magnification). NC, negative control; HC, healthy control; PBMC, peripheral blood mononuclear cell; LT, liver transplantation; H&E, hematoxylin and eosin; MT, Masson’s trichrome. *P < 0.05; ***P < 0.001; ****P < 0.0001.

Next, we generated an avatar model from tolerant patients to evaluate whether our model reflected real liver immunity. After generating the avatar models, the avatar’s liver tissue sections were stained with H&E, MT, and Sirius red at 7 weeks after injection of PBMCs. As depicted in **Figure 5A**, the tolerance group showed only mild inflammation and fibrosis compared to the rejection group and was similar to the HC group. Furthermore, confocal microscopic analysis revealed that the tolerance group had increased Treg cells and decreased Th17 cells compared to the rejection group, which agrees with the real liver immunity of tolerant patients (**Figure 5B**). These findings suggest that our patient-derived avatar model may reflect liver inflammation in each patient.

### Patient-Derived Avatar Model Treated With Tacrolimus and SD282 (Biguanide Derivatives): A Preclinical Model for Evaluating Treatment Responses

**Figure 6:**
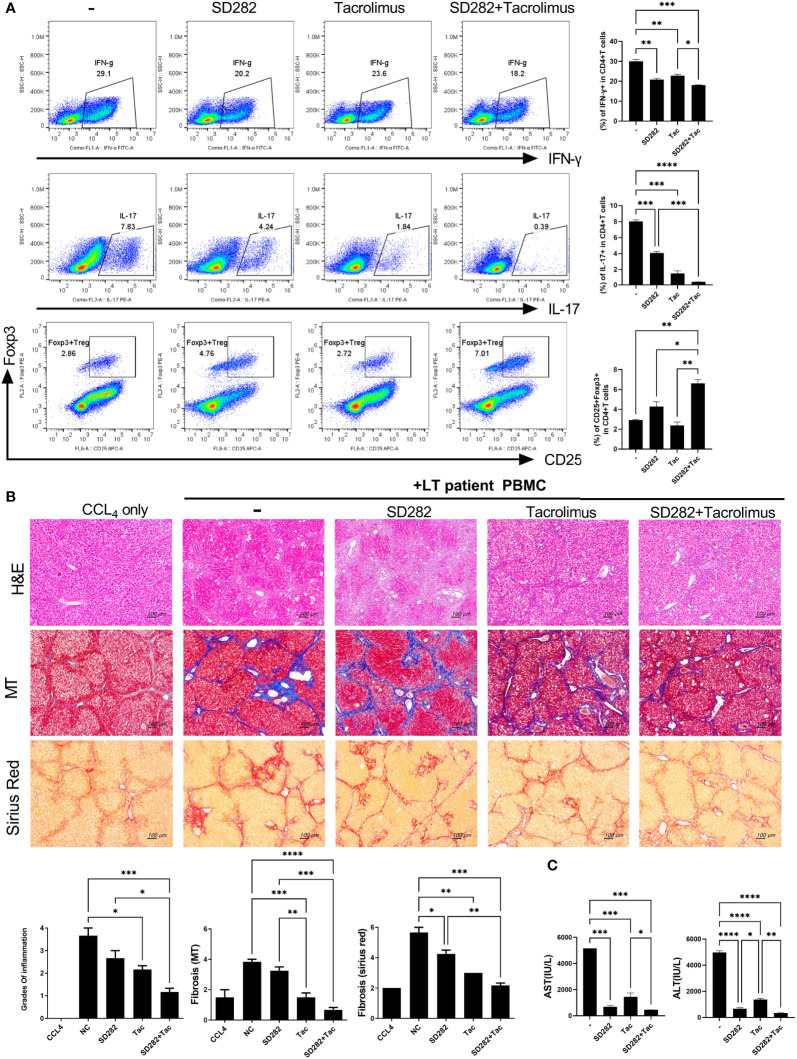
Change in T-cell subtypes (*in vitro* analysis) and liver histology of avatar models after combination treatments of tacrolimus and SD282 (biguanide derivatives). **(A)** Human CD4^+^ T cells from PBMCs were cultured in the presence or absence of SD282 or/and tacrolimus under the anti-CD3 stimulation condition for 3 days. The populations of Th1, Th17, and Foxp3^+^Tregs were analyzed by flow cytometry. **(B)** Changes in liver histology of avatar models after SD282 and tacrolimus combination therapy. Liver tissue sections were harvested from humanized mice at 7 weeks after adoptive transfer with PBMC preparations from liver transplantation patients. Humanized mice were orally administered vehicle (NC) or SD282 and/or tacrolimus once every day for 4 weeks. Representative images of H&E, MT, and Sirius red staining of paraffin-embedded mouse liver tissue sections are shown in the upper panel. Inflammation and fibrosis scores are shown in the lower panel. **(C)** Serum AST and ALT levels in humanized mouse model. Data are the means ± SEM of two replicates. ^*^ *P* < 0.05, ^**^ *P* < 0.01, ^***^ *P* < 0.001, ^****^ *P* < 0.0001. IHC, immunohistochemistry; PBMC, peripheral blood mononuclear cell; SD282, N-ethyl-N-(4-fluorophenyl) biguanide derivatives; H&E, hematoxylin and eosin; MT, Masson’s trichrome; AST, aspartate aminotransferase; ALT, alanine aminotransferase.

Finally, we performed a pilot study to evaluate the potential of our preclinical model for evaluating treatment responses in advance. To determine the effects of each treatment on inflammatory cells using PBMCs from stable LT patients, we first monitored the changes in immune markers such as Treg and Th17 cells after treatment with SD282 and/or tacrolimus under anti-CD3 stimulation. Compared with the results of tacrolimus monotherapy, the combination treatment of SD282 and tacrolimus led to a decrease in Th1 and Th17 cells and an increase in Treg cells (**Figure 6A**).

Thereafter, we generated avatar models using PBMCs from stable LT patients and treated the model with tacrolimus and/or SD282. The livers of avatar models treated with drugs were stained with H&E, MT, and Sirius red at 7 weeks after PBMC injection. Inflammation and fibrosis of the liver histology were alleviated after treatment with tacrolimus compared with that in the negative control. Moreover, combination treatment with tacrolimus and SD282 let to lower inflammation and less fibrosis than those in the negative control and each monotherapy group (**Figure 6B**). Similar trends were observed in the serum AST and ALT levels of each patient-derived mouse model, with significantly decreased AST and ALT levels after combination treatment compared to those in the negative control and monotherapy groups (**Figure 6C**). These results suggest that our model can be used for preclinical analysis of the effects of potential treatments on the liver of LT patients.

## Discussion

We developed a patient-derived avatar model to predict the liver immune microenvironment in LT patients. By engraftment of NSG mice with PBMCs from patients followed by several injections of hHSCs and CCl_4_, injected human inflammatory cells were CXCR3-dependently recruited into the liver and caused liver inflammation. According to the status of LT patients, the immune markers and liver histology of our models were quite different from and well-correlated with the patient’s immune status and liver histology. Interestingly, even stable post-LT patients had different liver immunities, and these differences were well-represented by our avatar model. Moreover, compared to monotherapy and negative control treatment, inflammation and fibrosis in the liver of avatar models were alleviated after combination treatment.

This avatar model was developed using NSG mice (NOD.Cg-*Prkdc* ^scid^ *IL2rg* ^tm1Wjl^/SzJ) engrafted with human PBMCs. These models are known to induce engraftment of T cells and have been used to evaluate T-cell rejection in human allografts, including skin engraftment (12, 23). Because this model could eventually develop lethal graft-versus-host disease (GVHD), inflammation of T cells was examined at 4–6 weeks before lethal GVHD (12); therefore, we visualized human cells and liver inflammation in avatar mice at 49 days after engraftment. The type engrafted only with PBMCs showed minimal inflammation, whereas our model infusing hHSCs and CCl_4_ after PBMC engraftment showed more severe inflammation in the liver without significant inflammation in the gut and spleen. Infused hHSCs can migrate to the site of injury, and the interaction of hHSCs with T cells perpetuate the immune response, leading to enhanced liver inflammation (24, 25). Moreover, infusion of a low dose of CCl_4_ and only half the dose for the conventional fibrosis model may help cause liver inflammation and fibrosis in engrafted human T cells. We infused only a small dose of CCl_4_ to prevent a conventional fibrosis while simultaneously enhancing inflammation and fibrosis in our avatar model (24, 25). Thus, stimulation of hHSCs and CCl_4_ amplified inflammation, which led to enhanced inflammation and immune response in the liver of our avatar models (26). However, although the immune response was enhanced by the combination of PBMCs, CCl_4_, and stellate cells, patient-derived T cells are the main contributor to the immune response, which can lead to differentiation of the inflammation of the liver of avatar models according to the immune homeostasis of LT patients.

Indeed, although all avatar models were injected with CCl_4_ and hHSCs, liver fibrosis and inflammation differed in accordance with the patient’s clinical status and were minimal in HCs. Avatar models from patients with rejection exhibited severe inflammation, whereas models from tolerant patients and HCs had mild inflammation in the blood and liver. As donor-reactive T cells are reduced in tolerant patients with increased levels of Treg cells (6, 27), their avatar models may show only mild inflammation similar to the liver immunity of HCs. Interestingly, in our study, avatar models from stable long-term LT patients were divided into severe and mild inflammation groups, which were correlated well with the patients’ real liver immunity. These results support that not all stable patients can achieve tolerance successfully because of alloreactive liver immunity in some patients (6, 28). Using this model, we evaluated whether liver immunity is stable, even in patients with long-term stability, which requires further verification. In our previous study, we identified patients who could minimize ISs or tolerance only after tapering ISs by analyzing PBMCs. To date, no reliable method has been developed to distinguish patients who are able to minimize ISs or tolerance before tapering ISs (6). Our developed model, representing and classifying the liver immunity and immune homeostasis of stable LT patients, can predict the prognosis of stable LT patients and facilitate a patient’s treatment plans, such as the minimization of an IS dose and the possibility of tolerance without liver biopsy and before tapering ISs.

It is possible that engrafted human T cells can migrate to the liver in the avatar model. To verify this mechanism, we examined the expression levels of chemokine ligands and receptors. In our avatar models, CXCR3-expressing Th17 cells and their ligands CXCL9, -10, and -11 were increased in the severe inflammation and rejection groups. Moreover, migration of Th17 cells toward activated LX-2 cells was significantly decreased in anti-CXCR-treated Th17 cells in *in vitro* migration analysis. Indeed, CXCR3 can activate and recruit T cells, such as Th17 cells, to the liver, causing liver inflammation and playing a crucial role in the development of viral-related liver inflammation and non-alcoholic steatohepatitis (29–33). In the context of CXCR3-dependent response in our results, CXCR3 may also play a pivotal role in the development of our new avatar model and contribute to different inflammations according to the status of LT patients. Recently, the anti-donor IFN-γ enzyme-linked immunosorbent spot assay was shown to have a predictive power for graft rejection after kidney transplantation. However, this assay has limitations in the accurate measurement of cytotoxic lymphocytes because of the possibility of its secretion by non-cytotoxic lymphocytes (34, 35). Meanwhile, the development of our new avatar model enables evaluation of the real liver immunity directly, even in stable LT patients *via* the CXCR3-dependent response of the model.

Furthermore, we examined changes in immunity of the avatar models after treatment with tacrolimus and/or SD282 (biguanide derivatives). We previously observed that the combination of SD282 and tacrolimus may increase immune cellular homeostasis (36). Our patient-derived avatar models showed that inflammation and fibrosis were alleviated, particularly after combination treatment with SD282 and tacrolimus. In onco-immunology, several mouse models have been developed and used to evaluate therapeutic agents or immune responses (11, 37). However, in the era of LT, there is an unmet need for preclinical models to evaluate the treatment response to various drugs and optimal dose of immunosuppressive drugs. Our results suggest that this model can be used to evaluate immune homeostasis in LT patients and as preclinical models for evaluating changes in the immune status following administration of various therapeutic drugs.

Our study had several limitations. First, it was a single-center study with a small number of patients. Second, we only investigated T-cell responses in our models. However, we created models from more than 40 patients with various clinical conditions and compared the histological findings and inflammatory cells in the blood of LT patients with those of paired avatar mice. We could not evaluate the B-cell response in our model mainly because of the low engraftment of human B cells from PBMCs in NSG mice (38). Moreover, considering the crucial role of T cells, including Treg cells, in the immune tolerance and rejection of LT patients (28, 39), our model is suitable for evaluating T-cell responses and predicting liver immunity without liver biopsy. Third, the immune response in this model may have been caused by various antigens including xenogeneic GVHD. There may be a concern that the results of this model do not reflect real liver immunity. However, in the clinical setting, it is difficult and unrealistic to elicit an immune response with the engraftment of liver tissue of stable liver LT patients in a humanized avatar model. Instead, by infusing the patient’s PBMCs followed by CCl_4_ and stellate cells, we observed the inflammation and immune response of the liver predicting the real liver immunity of LT patients in an *in vivo* animal model. However, avatar liver inflammation is caused not only by patient-derived T cells but also by a combination of PBMCs, CCl_4_, and stellate cells. Histologically confirmed differences in the immune microenvironments of stable LT patients were demonstrated using our avatar model, which correlated well with the patient’s histologic findings of the grafted liver.

In conclusion, we developed a patient-derived avatar model for predicting the actual liver immunity of LT patients without biopsy. Moreover, our results provide insights into the possible use of our model as a preclinical analysis tool for the potential treatment of the liver in LT patients. This new avatar model may provide clinicians with a direction for further treatment plans, including IS adjustment, and preclinical analysis for various therapeutic drugs.

## Data Availability Statement

The data are not publicly available due to ethical issues. Requests to access the datasets should be directed to Jong Young Choi, jychoi@catholic.ac.kr.

## Ethics Statement

The studies involving human participants were reviewed and approved by the institutional review board of Seoul St. Mary’s Hospital. The patients/participants provided their written informed consent to participate in this study. The animal study was reviewed and approved by the Animal Care and Use Committee of the Catholic University of Korea.

## Author Contributions

SKL, MJP, JYC, and MLC designed the experiments. MJP, JAB, JWC, and SYK performed the experiments. SKL, MJP, JWJ, SHB, SKY, HJC, YKY, JYC, and MLC analyzed and interpreted the data. SKL, MJP, JYC, and MLC wrote the article. JYC and MLC supervised the study. All authors contributed to the article and approved the submitted version.

## Funding

This study was supported by a grant from the Korea Health Technology R&D Project through the Korea Health Industry Development Institute (KHIDI), funded by the Ministry of Health & Welfare, Republic of Korea (grant number: HI15C3062). This work was also supported by the National Research Foundation of Korea (NRF) grant funded by the Korea government (MSIT) (No. 2020R1F1A1075816) and was supported by the Basic Science Research Program through the National Research Foundation of Korea (NRF) funded by the Ministry of Education (No. 2021R1I1A1A01050954).

## Conflict of Interest

The authors declare that the research was conducted in the absence of any commercial or financial relationships that could be construed as a potential conflict of interest.

## Publisher’s Note

All claims expressed in this article are solely those of the authors and do not necessarily represent those of their affiliated organizations, or those of the publisher, the editors and the reviewers. Any product that may be evaluated in this article, or claim that may be made by its manufacturer, is not guaranteed or endorsed by the publisher.

## Abbreviations

ALP, alkaline phosphatase; ALT, alanine aminotransferase; AST, aspartate aminotransferase; ELISPOT, enzyme-linked immunosorbent spot; EM, effector memory; GVHD, graft-versus-host disease; HAI, Histology Activity Index; HC, healthy controls; H&E, hematoxylin and eosin; HIS, human immune system; hHSCs, human hepatic stellate cells; IS, immunosuppressant; LT, liver transplantation; MIS, mild inflammation in stable; MT, Masson’s trichrome; PBMC, peripheral blood mononuclear cell; RAI, Rejection Activity Index; SEM, standard error of the mean; SIS, severe inflammation in stable; Th1, T helper 1; Th17, T helper 17; Treg, regulatory T.
