# Supplementary material for: Patient-Derived Avatar Mouse Model to Predict the Liver Immune Homeostasis of Long-Term Stable Liver Transplant Patients
Source: Front Immunol. 2022 Mar 28;13:817006. doi: 10.3389/fimmu.2022.817006 (PMC8995467; doi:10.3389/fimmu.2022.817006)
Supplement: Supplementary file 1 [file DataSheet_1.pdf]

Supplementary Material

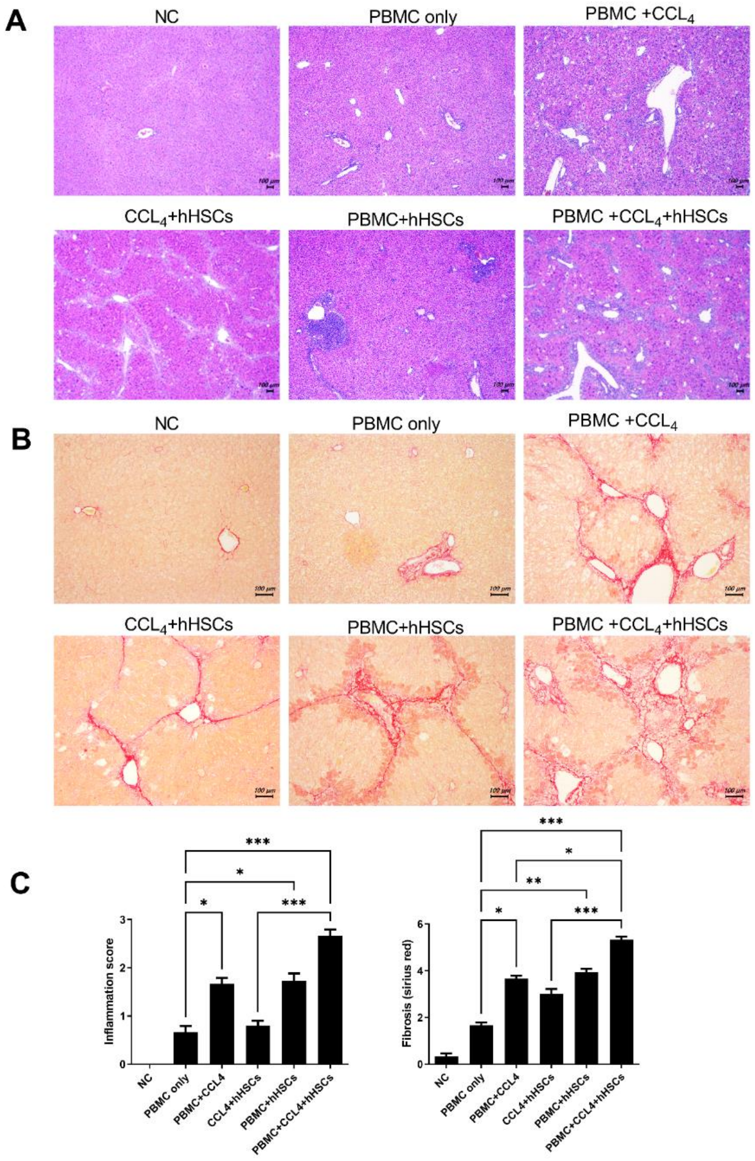

Supplementary Figure1. Comparison of liver histology of our avatar model and other experimental models. Representative liver histology stained with H&E (A) and Srius Red (B) in several conditions including our avatar model (injected with hHSCs and CCl4 after PBMC); model only injected with PBMC; model injected with PBMC followed by CCl4 injections; and model injected with PBMC followed by hHSCs injection (x100, original magnification). (C) The graph of

inflammation and fibrosis score. $^{*}P < 0.05$, $^{**}P < 0.01$, $^{***}P < 0.001$. H&E, hematoxylin and eosin; hHSCs, human hepatic stellate cells; PBMC, peripheral blood mononuclear cell.

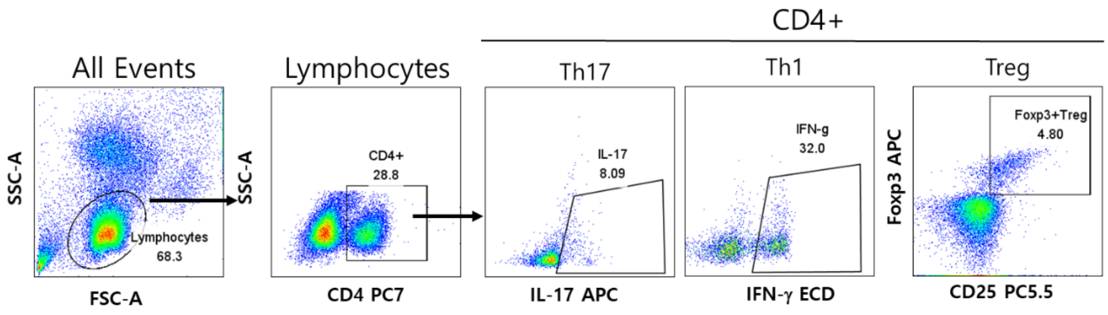

Supplementary Figure 2. Gating strategy for figure 2. The gating strategy used to evaluate by flow cytometry the T cell subsets from patient PBMC.

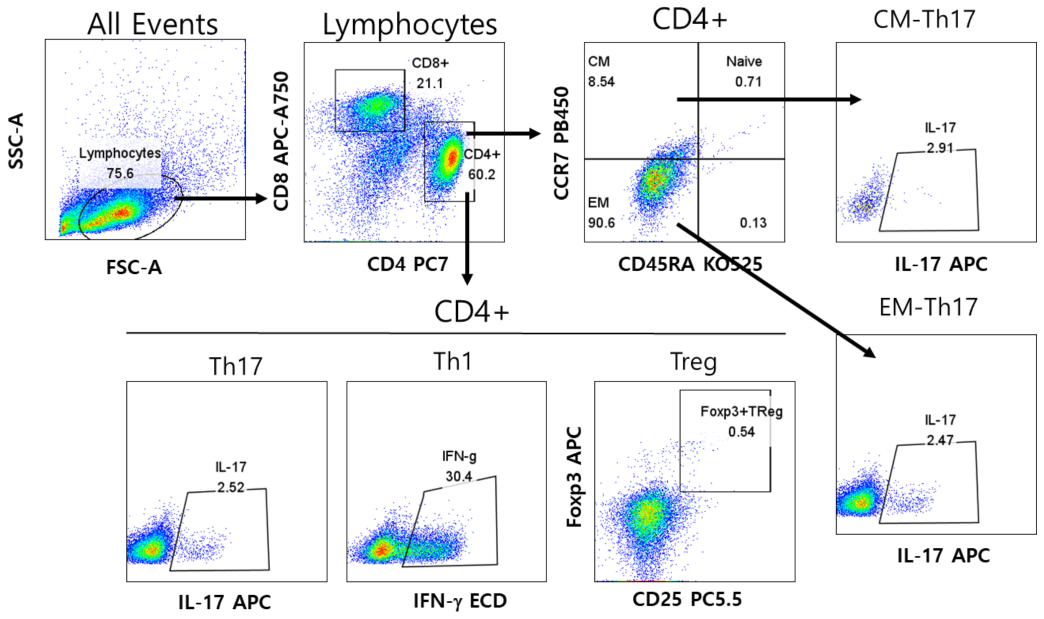

Supplementary Figure 3. Gating strategy for figure 3. The gating strategy used to evaluate by flow cytometry the T cell subsets in humanized mice.

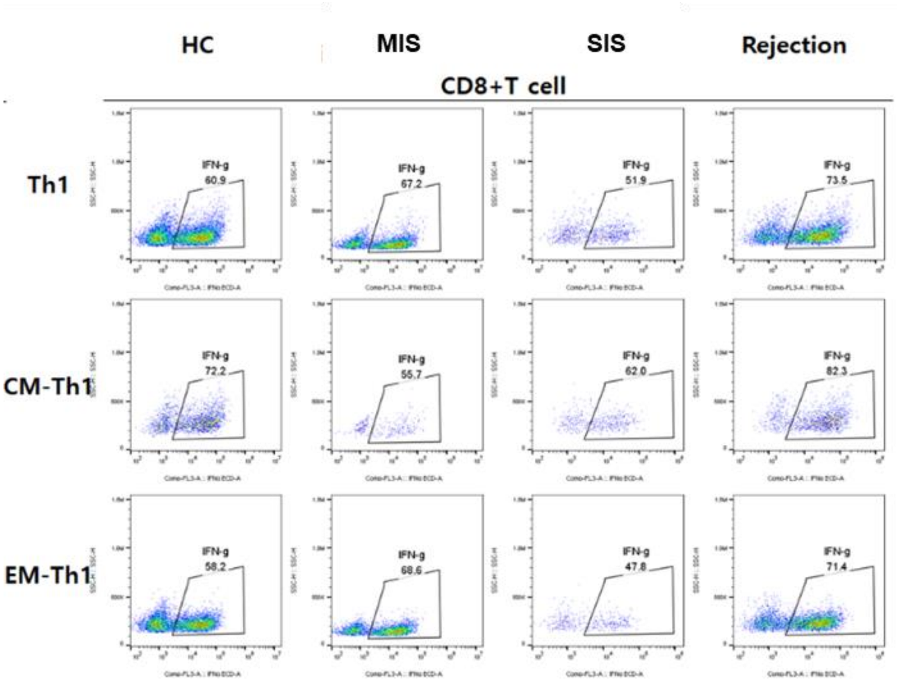

Supplementary Figure 4. The representative flow cytometric plot expressing the proportion of CD8+ T cell subtypes in spleen of each humanized mice group.

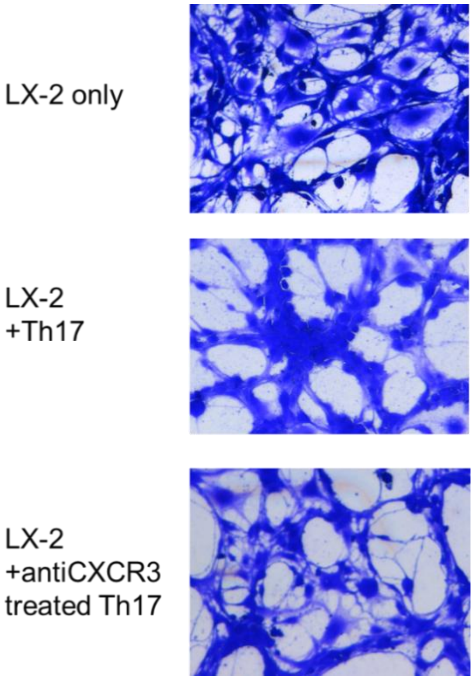

Supplementary Figure 5. In vitro migration of Th17 cells toward the activated LX-2 cells

Supplementary table 1. Antibodies for flow cytometry.

Antibody	Clonality	Clone	Fluorophore	Host
Human CD4	Monoclonal	RPA-T4	PE/Cy7	Mouse
Human CD8	Monoclonal	SK1	APC-Cy7	Mouse
Human CD25	Monoclonal	BC96	APC	Mouse
Human CD197(CCR7)	Monoclonal	150503	BV 421	Mouse
Human CD45RA	Monoclonal	HI100	BV510	Mouse
Human Foxp3	Monoclonal	236A/E7	APC	Mouse
Human IL-17	Monoclonal	eBio64DEC17	APC	Mouse
Human IFN-g	Monoclonal	B27	PE-CF594	Mouse

Supplementary Table 2. Antibodies for immunohistochemistry

Antibody	Clonality	Clone	Host
CD4	Monoclonal	EPR6855	Rabbit
CD8	polyclonal		Rabbit
CD19	Monoclonal	EPR5906	Rabbit
CK19	Monoclonal	EP1580Y	Rabbit
IL-17	Polyclonal		Goat
FOXP3	Monoclonal	236A/E7	Mouse
CXCL9	Polyclonal		Rabbit
CXCL10	Polyclonal		Rabbit

CXCL11	Polyclonal		Rabbit
CCL2	polyclonal		Rabbit
CCL3	polyclonal		Goat
CCR2	Monoclonal	2D8	Mouse

Supplementary Table 3. Primary and secondary antibodies for confocal

1st Antibody	Clonality	Clone	Host
CD4	Monoclonal	EPR6855	Rabbit
IL-17	Polyclonal		Rabbit
CXCR3	Monoclonal	H-1	Rabbit
Total STAT3	Monoclonal	7D1	Rabbit
pSTAT3(Y705)	Monoclonal	B-7	Goat
CD4 - Alexa Fluor® 594	Monoclonal	GK1.5	Mouse
IL-17	Polyclonal		Rabbit
CXCR3	Monoclonal	49801	Rabbit
CD25	Monoclonal	IL2R.1	Rabbit
Foxp3	Polyclonal		Rabbit
**2nd Antibody**	**Clonality**	**Clone**	**Host**
anti-Rabbit IgG (H+L)-Alexa Fluor® 488	Polyclonal	EPR6855	Donkey
anti-Goat IgG (H+L)-PE	Polyclonal		Donkey
Anti-Mouse IgG(H+L)-Alexa Fluor® 647	Polyclonal	H-1	Goat

Anti-Mouse IgG(H+L)-Alexa Fluor® 647	Polyclonal	7D1	Goat
Anti-Mouse IgG(H+L)-FITC	Polyclonal	B-7	Goat
Anti-goat IgG-APC	Polyclonal	GK1.5	Donkey
Anti-Mouse IgG(H+L)-FITC	Polyclonal		rabbit
Anti-Mouse IgG(H+L)-Alexa Fluor® 647	Polyclonal	49801	Goat
Anti-Rabbit IgG(H+L)-PE	Polyclonal	IL2R.1	Goat
